# Supplementary material for: Functional Aspects of Early Light-Induced Protein (ELIP) Genes from the Desiccation-Tolerant Moss Syntrichia caninervis
Source: Int J Mol Sci. 2020 Feb 19;21(4):1411. doi: 10.3390/ijms21041411 (PMC7073071; doi:10.3390/ijms21041411)
Supplement: Supplementary file 1 [file ijms-21-01411-s001.pdf]

International Journal of Molecular Sciences

MDPI

Table S1. Properties of 6 ScELIPs Unigenes.

	Predicted Number of Amino Acid	Predicted pI	Predicted Molecular Aass (kDa)	Conserved Domain
13021	212	5.58	23.2	Chloroa_b-bind
40120	236	9.09	25.5	Chloroa_b-bind
40121	236	9.33	25.7	Chloroa_b-bind
68225	207	9.57	21.7	Chloroa_b-bind
16576	94	10.39	10.2	Chloroa_b-bind
8044	94	6.71	10.4	Chloroa_b-bind

Table S2. Sequences information for construction of the phylogenetic tree.

	Gene	Amino Acid	Accession Number
AtELIP1	*ELIP1*	151	U89014
AtELIP2	*ELIP2*	152	Z97336
Syntrichia ruralis-A	*ELIPa*	212	AY034890
Syntrichia ruralis-B	*ELIPb*	224	AY034891
Setaria italica	*ELIP*	181	XP_004955632
Zea mays	*ELIP*	192	ACG46269
Panicum miliaceum	*ELIP*	197	RLN34913.1
Oryza sativa	*ELIP*	187	BAD30330
Brachypodium distachyon	*ELIP*	193	XP_010228291.1
Dendrobium catenatum	*ELIP*	192	XP_020674324
Apostasia shenzhenica	*ELIP*	196	PKA64958.1
Elaeis guineensis	*ELIP*	189	XP_010923519.1
Phoenix dactylifera	*ELIP*	193	XP_008786056.1
Solanum tuberosum	*ELIP*	197	XP_006363471
Medicago truncatula	*ELIP*	197	XP_013469748.1
Gossypium raimondii	*ELIP*	193	XP_012487200.1
Morus notabilis	*ELIP*	195	XP_010107710
Capsella rubella	*ELIP*	196	XP_006298609.1
Brassica rapa	*ELIP*	191	XP_009148406.1
Selaginella moellendorffii	*ELIP*	197	XP_024536558
Sphagnum fallax	*ELIP*	253	Sphfalx0001s0155.1
Onoclea sensibilis	*ELIP*	230	AAB25012
Marchantia polymorpha	*ELIP*	227	PTQ40288
Physcomitrella patens ELIP12	*ELIP12*	225	A9TAX2
Physcomitrella patens ELIP8	*ELIP8*	325	EDQ69484
Physcomitrella patens ELIP11	*ELIP11*	110	A9S3S8

Physcomitrella patens ELIP7	*ELIP7*	140	A9TL19
Physcomitrella patens ELIP6	*ELIP6*	135	A9RSF4
Physcomitrella patens ELIP10	*ELIP10*	220	A9SZ27
Physcomitrella patens ELIP4	*ELIP4*	249	A9TIL7
Physcomitrella patens ELIP9	*ELIP9*	225	A9T6B6
ScELIP1	*ScELIP1*	236	KM363766
ScELIP2	*ScELIP2*	207	KM363767

Table S3. Primer information for RT-qPCR of high light treatment of plantlets.

Gene		Sequence	GeneBank Accession	
OHP2	Forward	TCTTCGACAGCTTCGTCGTCT	NM_103122	one helix protein
	Reverse	GAGGCTCCCGGAGAGTAGGA		
OHP	Forward	TGAGCTCGTCGCCGTTATCT	NM_120290.3	one helix protein
	Reverse	GCGGCTCTGACAACGAAAGG		
SEP2	Forward	TGGCGATTTAGCCGAGAACGG	NP565524	stress enhanced protein 2
	Reverse	CACCATCTCCGCCGTCCTTT		
LHCB4.2	Forward	ATCGACGCCGTTTCAGCCAT	NM_111728	PS II CP29 antenna protein
	Reverse	GCCAAGAGTGGCGAGCATTG		
LHCA2	Forward	CTGTCTCCAGGCCAGATGCG	NM_116012	PS I antenna protein
	Reverse	CACCAGGGAGGCTACCGTCT		
PSBS	Forward	GGGAGCCATTGGAGCTCTCG	NM_103552	PS II CP22 antenna protein
	Reverse	GAGACCGAGGGCAGATCGGA		
PSBD	Forward	TGATGGGAGTCGCCGGTGTA	NP_051054	PS II D2 protein
	Reverse	AAGCGGTTAGCGGTGACCAT		
ScELIP1	Forward	GGCTATGCTGGGATTCGTGT	KM363766	
	Reverse	GACGCCAGGAACCAGATCAA		
ScELIP2	Forward	GCAATGATCGGACTTGTCGC	KM363767	
	Reverse	ACGGATTGTTCACAGCAGGT		
α-TUB	Forward	GATGTACCGTGGTGATGTC		
	Reverse	GAGCCTCTGAAAATTCTCC		

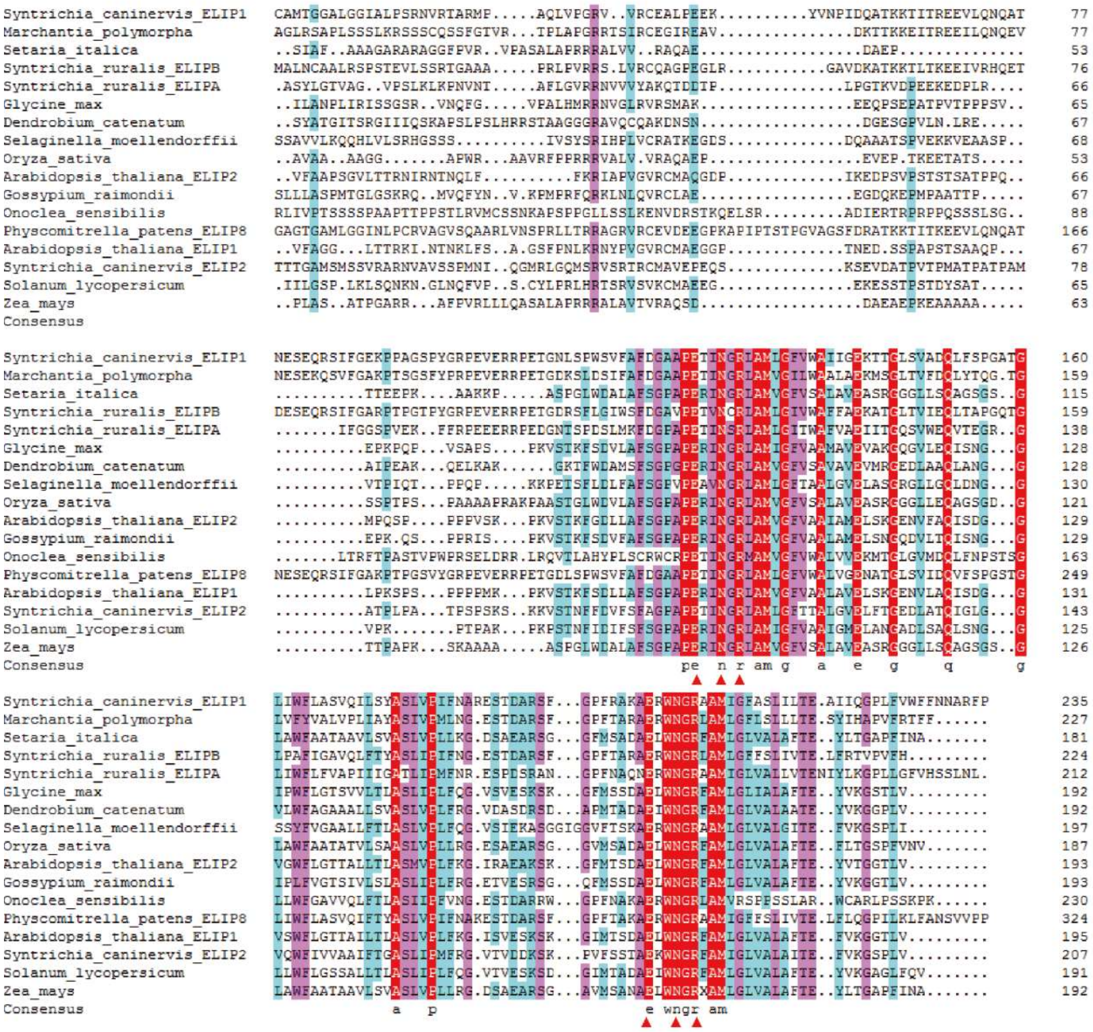

Figure S1. The amino acid sequences alignment of ELIPs by MEGA 7.0. *ScELIP1* and *ScELIP2* have conserved amino acids (triangular symbol) to known ELIP proteins.

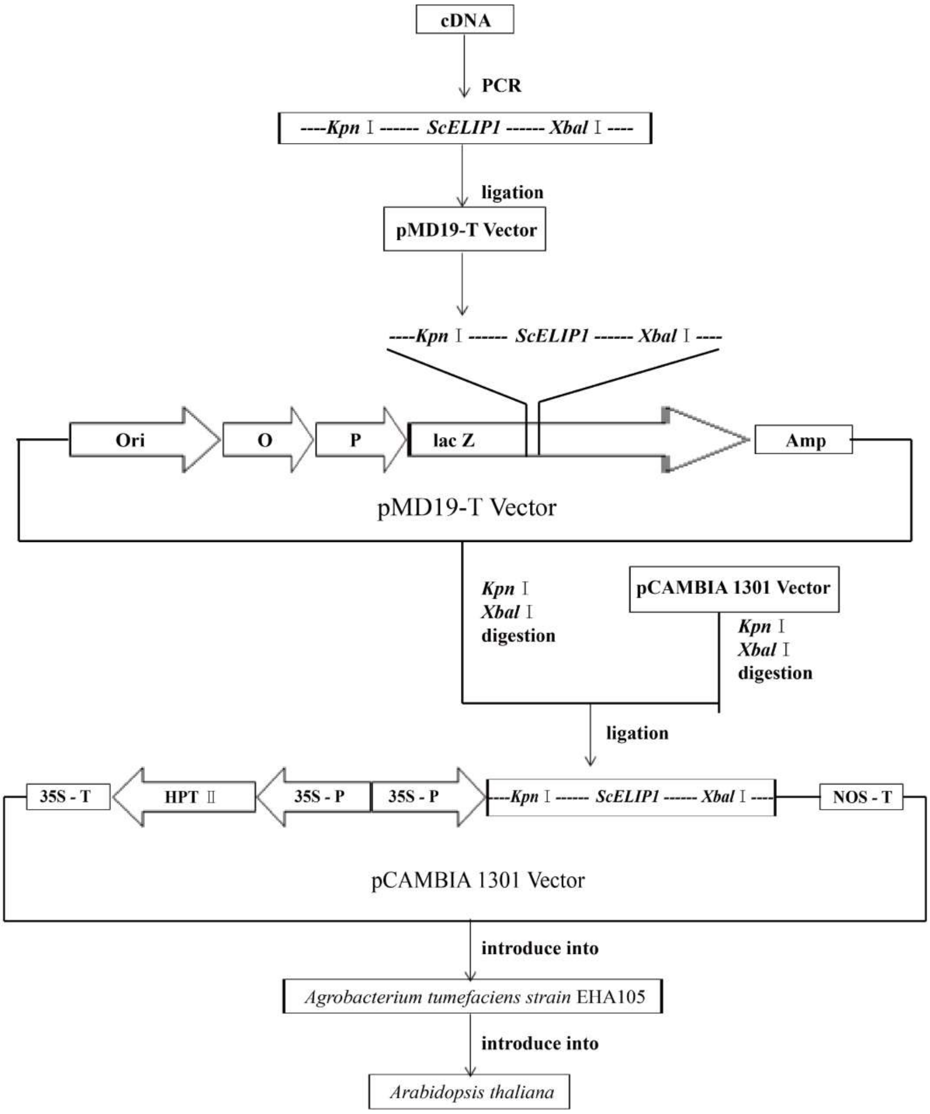

Figure S2. 35S-*ScELIP1* vector construction strategy.

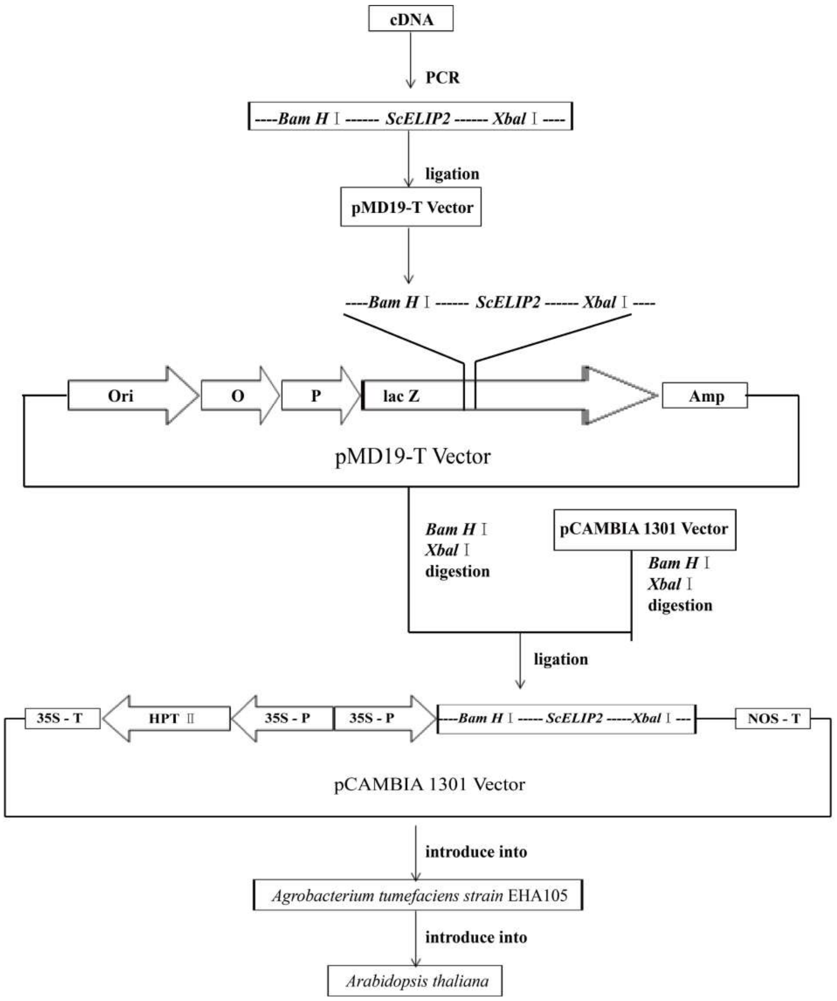

Figure S3. 35S-*ScELIP2* vector construction strategy.

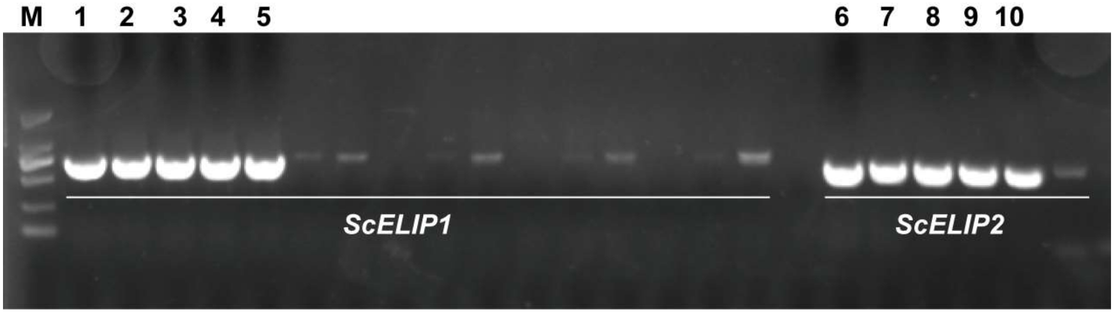

Figure S4. Verification of transgenetic Agrobacterium by bacterium PCR. Lane 1-5 positive transgenic clones for *ScELIP1*, lane 6-10 positive transgenic clones for *ScELIP2*. Lane 1 and 6 Agrobacterium used for transforming *Atelip* mutant.

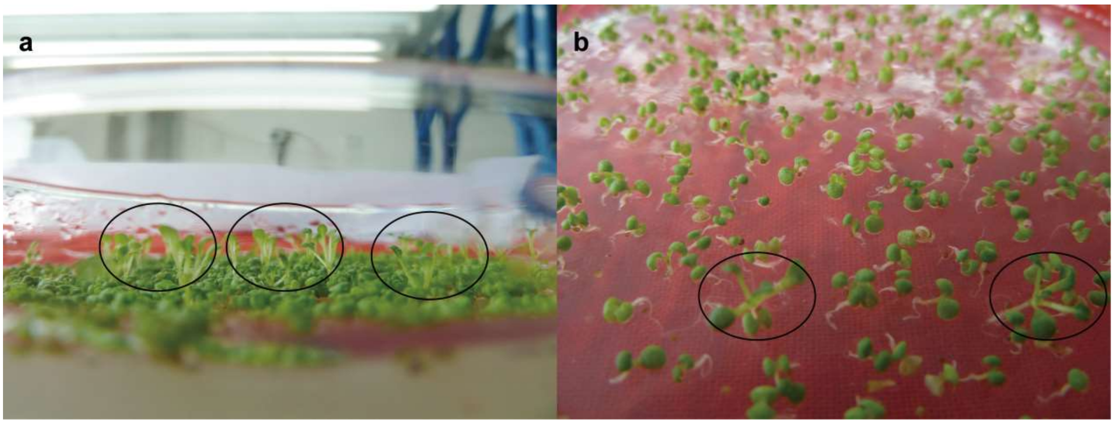

Figure S5. Hygromycin selection of transgeneic *Arabidopsis thaliana*. The plantlets in the circles are positive transgenic lines which have emergent green leaves which stay green, the negative plants have cotyledon emergence but subsequently turn yellow or white after ten days.

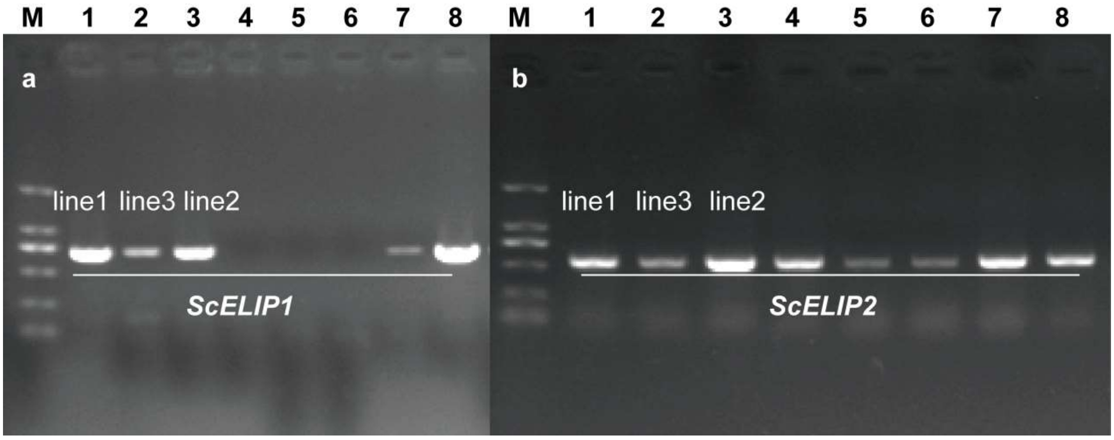

Figure S6. RT-PCR validation of T3 transgenic lines.

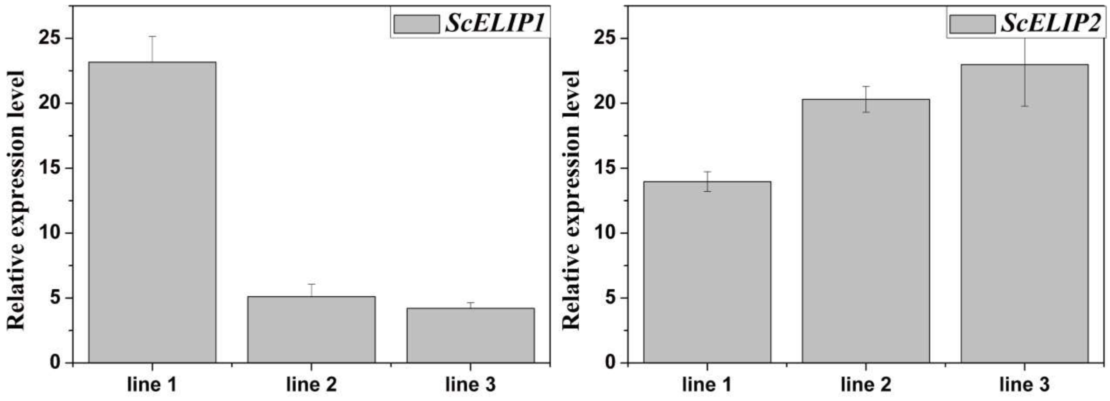

Figure S7. Quantitative real time PCR expression level analysis of *ScELIP1* and *ScELIP2* transgenic lines. Error bars represent the SD of three biological repeats. Relative expression values were obtained from $2^{-\triangle\triangle Ct}$ comparing WT and the transgenic lines with the *Atelip* mutant respectively.

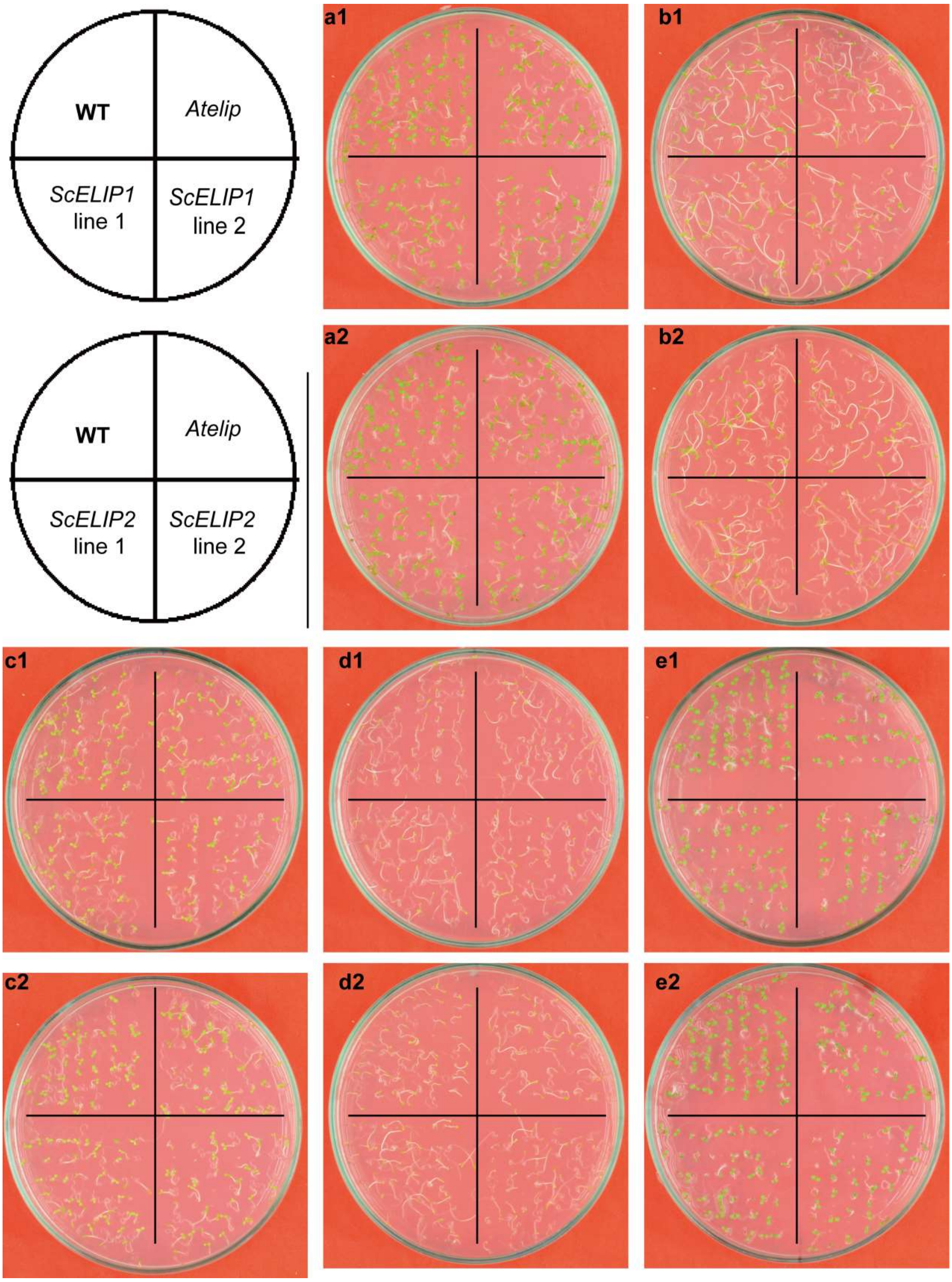

Figure S8. Seed germination phenotypes for Col-0 WT, *Atelip* mutant and *35S-ScELIP* lines under different light treatments. Images, labeled a-e, depict the phenotypic response of the four genotypes to the various light treatments. Control condition (**a1**,**a2**); Red light alone (**b1**,**b2**); Blue light alone (**c1**,**c2**); UVB alone (**d1**,**d2**); High light alone (**e1**,**e2**).

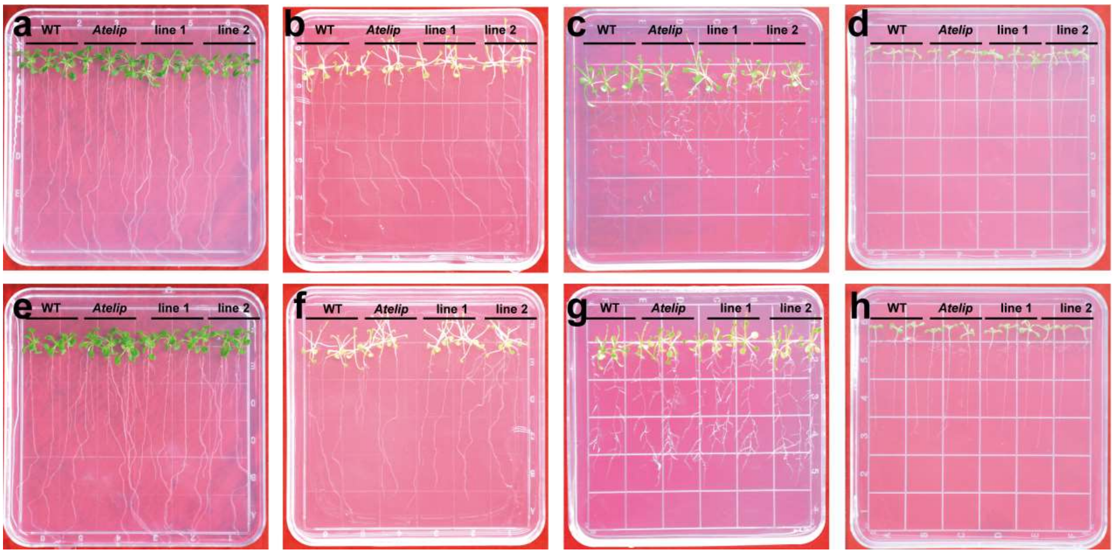

Figure S9. Phenotypic response of Col-0 WT, *Atelip* mutant and *35S-ScELIP1* and *35S-ScELIP2* after seven days of exposure to different light treatments. Row 1, WT, *Atelip* mutant, *35S-ScELIP1* transgenic lines. Row 2, WT, *Atelip* mutant, *35S-ScELIP2* transgenic lines. Control conditions (**a**, **e**), Red light alone (**b**, **f**), Blue light alone (**c**, **g**), UVB alone (**d**, **h**).

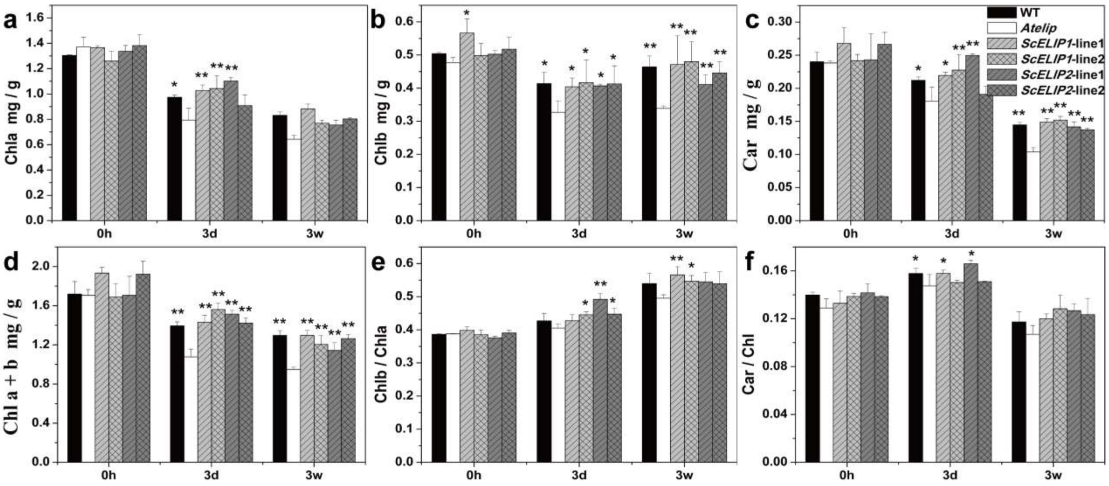

Figure S10. The change of chlorophyll content of WT, *Atelip* and transgenic lines at 0 h, 3 days and 3 weeks high light stress (1000 μmol/m²/s). Error bars represent the SD of three biological repeats. *, P < 0.05; **, P < 0.01. P-values were obtained from Dunnett's T3 test comparing WT and the transgenic lines with the *Atelip* mutant respectively.

© 2020 by the authors. Licensee MDPI, Basel, Switzerland. This article is an open access article distributed under the terms and conditions of the Creative Commons Attribution (CC BY) license (http://creativecommons.org/licenses/by/4.0/).
